# Inferring Nonlinear Neuronal Computation Based on Physiologically Plausible Inputs

**DOI:** 10.1371/journal.pcbi.1003143

**Published:** 2013-07-18

**Authors:** James M. McFarland, Yuwei Cui, Daniel A. Butts

**Affiliations:** Department of Biology and Program in Neuroscience and Cognitive Science, University of Maryland, College Park, Maryland, United States of America; University of Tübingen and Max Planck Institute for Biologial Cybernetics, Germany

## Abstract

The computation represented by a sensory neuron's response to stimuli is constructed from an array of physiological processes both belonging to that neuron and inherited from its inputs. Although many of these physiological processes are known to be nonlinear, linear approximations are commonly used to describe the stimulus selectivity of sensory neurons (i.e., linear receptive fields). Here we present an approach for modeling sensory processing, termed the Nonlinear Input Model (NIM), which is based on the hypothesis that the dominant nonlinearities imposed by physiological mechanisms arise from rectification of a neuron's inputs. Incorporating such ‘upstream nonlinearities’ within the standard linear-nonlinear (LN) cascade modeling structure implicitly allows for the identification of multiple stimulus features driving a neuron's response, which become directly interpretable as either excitatory or inhibitory. Because its form is analogous to an integrate-and-fire neuron receiving excitatory and inhibitory inputs, model fitting can be guided by prior knowledge about the inputs to a given neuron, and elements of the resulting model can often result in specific physiological predictions. Furthermore, by providing an explicit probabilistic model with a relatively simple nonlinear structure, its parameters can be efficiently optimized and appropriately regularized. Parameter estimation is robust and efficient even with large numbers of model components and in the context of high-dimensional stimuli with complex statistical structure (e.g. natural stimuli). We describe detailed methods for estimating the model parameters, and illustrate the advantages of the NIM using a range of example sensory neurons in the visual and auditory systems. We thus present a modeling framework that can capture a broad range of nonlinear response functions while providing physiologically interpretable descriptions of neural computation.

## Author Summary

Sensory neurons are capable of representing a wide array of computations on sensory stimuli. Such complex computations are thought to arise in large part from the accumulation of relatively simple nonlinear operations across the sensory processing hierarchies. However, models of sensory processing typically rely on mathematical approximations of the overall relationship between stimulus and response, such as linear or quadratic expansions, which can overlook critical elements of sensory computation and miss opportunities to reveal how the underlying inputs contribute to a neuron's response. Here we present a physiologically inspired nonlinear modeling framework, the ‘Nonlinear Input Model’ (NIM), which instead assumes that neuronal computation can be approximated as a sum of excitatory and suppressive ‘neuronal inputs’. We show that this structure is successful at explaining neuronal responses in a variety of sensory areas. Furthermore, model fitting can be guided by prior knowledge about the inputs to a given neuron, and its results can often suggest specific physiological predictions. We illustrate the advantages of the proposed model and demonstrate specific parameter estimation procedures using a range of example sensory neurons in both the visual and auditory systems.

## Introduction

Sensory perception in the visual and auditory systems involves the detection of elemental features such as luminance and sound intensity, and their subsequent processing into more abstract representations such as “objects” that comprise our perception. The neuronal computations performed during such sensory processing must be nonlinear in order to generate more complex stimulus selectivity, such as needed to encode the conjunction of multiple sensory features [1]–[3] as well as to develop invariance to irrelevant aspects of the raw sensory input [4], [5]. While these computations can appear inscrutably complex, they are necessarily constructed from the underlying neural circuitry, which exhibits several well-known and relatively straightforward nonlinear properties.

Nevertheless, characterizations of sensory neurons still typically rely on the assumption of linear stimulus processing, which is often implicit in standard approaches such as spike-triggered averaging and – more recently – generalized linear models (GLMs) [6]–[8]. While such descriptions can often provide good predictions of the neuronal response [9]–[11], they necessarily leave out the nonlinear elements of neuronal processing that likely play a major role in building the sensory percept.

Unfortunately, the space of possible nonlinear models is not bounded. While one might be inclined to incorporate details of the system and circuitry in question, more complicated models require more data for parameter estimation, and often involve poorly behaved or intractable optimization problems. As a result, practical nonlinear modeling approaches must make assumptions that limit the space of functions considered by restricting to a defined set of nonlinear interactions.

Several different approaches have been developed in this regard. The most common is to identify a low dimensional “feature space” to which the neuron is sensitive, with the assumption that its firing rate depends on a nonlinear function applied only to these stimulus features. Prominent examples of this approach include spike-triggered covariance (STC) analysis [12], [13], which uses the covariance of the stimuli that elicit spikes, and information-theoretic approaches such as maximally informative dimensions (MID) analysis [14] and iSTAC [15]. With the subspace determined, other methods can be used to estimate a nonlinear mapping between the projection of the stimulus onto this low dimensional feature space and the firing rate [13]–[19].

A second general approach is to assume the form of nonlinearities present, most commonly based on a second-order approximation of the nonlinear stimulus-response relationship, as with the Wiener-Volterra expansion [20]–[24], and more recent versions cast in a probabilistic context [25]–[28]. This category might also encompass neural network approaches, which characterize the stimulus-response relationship in terms of a set of fixed nonlinear basis functions, using either generic network elements [29], [30] or more specific nonlinear models of upstream sensory processing [31], [32].

A final commonly used approach assumes that relevant nonlinearities can be captured by directly augmenting the linear model to account for specific response properties, such as the addition of refractoriness to account for neural precision [7], [8], [33]–[35], feedback terms that account for adaptation to contrast [36]–[38], and other nonlinearities to capture response properties such as sensitivity to stimulus intensity and local context [25].

Here, we present a probabilistic modeling framework inspired by all of these approaches, the ‘Nonlinear Input Model’ (NIM), which limits the space of nonlinear functions by assuming that nonlinearities in sensory processing are dominated by spike generation, resulting in both rectification of the inputs to the neuron, as well as rectification of the neuron's output. By assuming a neuron's inputs are rectified, the NIM implicitly describes neuronal processing as a sum over excitatory and inhibitory inputs, which is increasingly being seen as an important factor in sensory processing [39]–[43]. The NIM expands directly on the GLM framework, and is able to utilize recent advances in the statistical modeling of neural responses [7], [8], [17], [44], [45], including the ability to model spike-refractoriness [7],[8] and multi-neuron correlations [8],[44].

As we show here, this results in a parsimonious nonlinear description of a range of neurons in both the visual and auditory systems, and has several advantages over previous approaches. Because of its relatively simple model structure, parameter estimation is well-behaved and makes efficient use of the data, even when the number of relevant inputs is large and/or the stimulus is high-dimensional. Importantly, because its form is based on an integrate-and-fire neuron, model selection and parameter estimation can be guided by specific knowledge about the inputs to a given neuron, and the elements of the resulting model can often be related to specific physiological predictions. The NIM thus provides a powerful and general approach for nonlinear modeling that complements other methods that rely on more abstract formulations of nonlinear computation.

## Results

### Nonlinear combination of multiple inputs: ON-OFF retinal ganglion cells

Perhaps the greatest success of linear models is in the retina, where it has been used primarily to describe the spike responses of retinal ganglion cells (RGCs) [10], [11], [16]. For a given RGC, estimating the components of the linear model typically involves measuring its spiking response to a noise stimulus, and then computing the average stimulus that preceded its spikes: the spike-triggered average (STA). The STA linear filter can produce very good response predictions for typical RGCs under stationary stimulus conditions, but clearly fails for ON-OFF cells (commonly found in rodents), which respond to both increases and decreases of light intensity [46]–[49]. This failure for ON-OFF cells occurs simply because the STA identifies only a single stimulus dimension, and averages out the opposing stimulus features that evoke ON and OFF responses.

**Figure 1:**
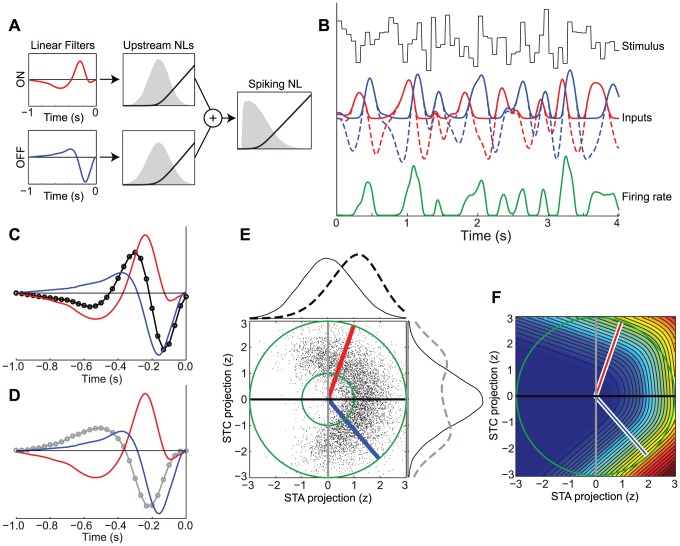
ON-OFF RGC simulation. **A**) Schematic showing the ON (top, red) and OFF (bottom, blue) inputs to the simulated ON-OFF RGC. The temporal filters (left) process the stimulus, and the upstream nonlinearities (black, middle) are then applied to the filter outputs. The sum of the two inputs is then passed through the spiking nonlinearity (black, right). The distributions of the stimulus filtered by the ON and OFF pathways, as well as the distribution of their summed input to the simulated neuron are shown as gray shaded regions. **B**) A simulation showing how the response to a 15 Hz (Gaussian) white noise stimulus is constructed. The stimulus (black) is filtered by the ON and OFF temporal kernels (dashed red and blue), and then transformed by the upstream nonlinearities (solid red and blue). The resulting instantaneous firing rate (green) is given by the sum of these inputs passed through the spiking nonlinearity. **C**) The STA (black) resembles the average of separate ON (red) and OFF (blue) filters of the generative model. **D**) Similar to panel C, the first STC filter (gray) resembles a mixture of the ON and OFF filters. **E**) Stimuli eliciting spikes (black dots) are projected onto the two-dimensional subspace spanned by the STA and first STC filter, shown in units of z-score. The distributions of stimuli corresponding to spikes (dashed lines on top and left) are compared to the marginal stimulus distributions (solid), demonstrating a systematic bias along the STA (horizontal) axis, and an increased variance along the STC (vertical) axis. The true ON and OFF filters (red and blue) are also contained in the STA/STC subspace, as indicated by the red and blue lines lying on the unit circle (green). The inner green circle has a radius of one standard deviation. **F**) The neuron's firing rate as a function of the stimulus projected into the 2-D STA/STC subspace (shaded color depicts firing rate: increasing from blue to red).

To explore this situation, we construct a basic model of an ON-OFF RGC, which receives separate ON and OFF inputs (Fig. 1A). If these two inputs were to combine linearly, their effect would be identical to that of a single input generated by the sum of the two stimulus filters, i.e., (**s**·**k**_ON_)+(**s**·**k**_OFF_) = **s**·(**k**_ON_+**k**_OFF_) = **s**·**k**_SUM_. Here the stimulus **s** at a particular time is represented as a vector (which in general includes time-lagged elements to account for stimulus history) such that the operation of a linear filter **k** is given by a dot product. Because of the averaging implicit in linear processing, a nonlinear transformation must be applied to each input in order to enable the model ON-OFF neuron to respond to both types of stimuli: i.e., *f*(**s**·**k**_ON_)+*f*(**s**·**k**_OFF_). These *f*(.) are taken to be rectifying functions (Fig. 1A), as seen experimentally [50], [51], and as modeled in [52]–[54]. As a result, the response of the neuron to increases or decreases of luminance is dominated by the ON or OFF pathways respectively (Fig. 1B), producing a response that is selective to both ON and OFF stimulus dimensions. As expected, the STA (Fig. 1C) for this neuron does not match either the ON or OFF stimulus filters, but rather reflects their average.

Thus, this is a clear example where nonlinear characterization is necessary to capture the RGC's stimulus selectivity. One such approach that has been applied to ON-OFF cells is spike-triggered covariance (STC) analysis [49], [55], which identifies stimulus dimensions along which the variance of the spike-triggered ensemble is either increased or decreased relative to the stimulus distribution [12], [13]. For the example neuron in Fig. 1, STC analysis identifies a stimulus dimension along which the variance of the spike-triggered ensemble is expanded (Figs. 1D, E). While neither the STA nor STC filters correspond to the true ON or OFF filters, together they define a stimulus subspace that contains the true filters (Fig. 1E).

Given the dimensionality reduction achieved in determining the STC subspace (or with other subspace identification methods), it is possible in principle to completely characterize the neural response function, i.e., *r* = *F*[**k**_1_·**s**, **k**_2_·**s**]. In two-dimensions, such as in this example, this nonlinear mapping from the subspace to a firing rate can be estimated non-parametrically [18],[56] given enough data, and potentially approximated in higher dimensions [13], [15], [18], [19], [57].

However, even if accurate estimation of this nonlinear mapping were possible, such functions are difficult to interpret, even when arising from the conjunction of simpler components. For example, in our simulated ON-OFF RGC, neither the STA/STC filters themselves nor the measured nonlinear mapping make it clear that the response is generated from separate inputs with relatively straightforward nonlinearities.

This example thus motivates the modeling framework that we present here, the Nonlinear Input Model (NIM), which describes a neuron's stimulus processing as a sum of nonlinear inputs, following the structure of the generative model shown in Fig. 1. Below, we first present procedures for estimating the parameters of the NIM before demonstrating its ability to recover the inputs to the ON-OFF RGC, as well as its application to a range of other simulated and measured data from both visual and auditory brain areas.

### Parameter estimation for the Nonlinear Input Model (NIM)

**Figure 2:**
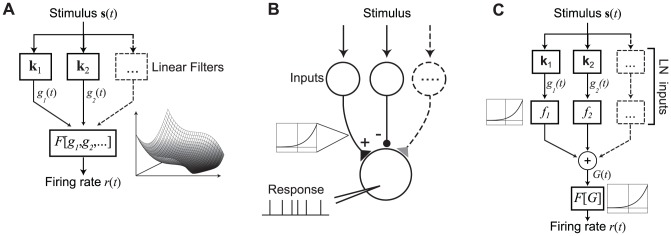
Schematic of LN and NIM structures. A) Schematic diagram of an LN model, with multiple filters (k_1_, k_2_, …) that define the linear stimulus subspace. The outputs of these linear filters (*g*_1_, *g*_2_, …) are then transformed into a firing rate prediction *r*(*t*) by the static nonlinear function *F*[*g*_1_,*g*_2_,…], depicted at right for a two-dimensional subspace. Note that while the general LN model thus allows for a nonlinear dependence on multiple stimulus dimensions, estimation of the function *F*[.] is typically only feasible for low (one- or two-) dimensional subspaces. B) Schematic illustration of a generic neuron that receives input from a set of ‘upstream’ neurons that are themselves driven by the stimulus s. Each of the upstream neurons provides input to the model neuron that is generally rectified due to spike generation (inset at left), and thus is either excitatory or inhibitory. The model neuron then integrates its inputs and produces a spiking output. C) Block diagram illustrating the structure of the NIM, based on (B). The set of inputs are represented as (one-dimensional) LN models, with a corresponding stimulus filter k_i_, and “upstream nonlinearity” *f_i_*(.). These inputs are then linearly combined, with weights *w*_i_, and fed into the spiking nonlinearity *F*[.], resulting in the predicted firing rate *r*(*t*). The NIM thus has a ‘second-order LN’ structure (or LNLN), with the neuron's own nonlinear processing shaped by the LN nature of its inputs.

The computational challenges associated with parameter estimation are a significant barrier to the successful development and application of nonlinear models of sensory processing. In the standard linear-nonlinear (LN) model, the neuron's response is modeled by an initial stage of linear stimulus filtering, followed by a static nonlinear function (“spiking nonlinearity”) that maps the output to a firing rate (Fig. 2A). The more recent adaptation of probabilistic models based on spike train likelihoods, such as in the Generalized Linear Model (GLM) [6]–[8], allows for integration of other aspects of neuronal processing into the linear stimulus-processing framework, and can be used to model nonlinear stimulus processing through predefined nonlinear transformations [31], [32], [58]. Importantly, this approach also provides a foundation for parameter estimation for the NIM.

A principal motivation for the NIM structure is that if the neuronal output at one level is well described by an LN model, downstream neurons will receive inputs that are already rectified (or otherwise nonlinearly transformed). Thus, we use LN models to represent the inputs to the neuron in question, and the neuron's response is given by a summation over these LN inputs followed by the neuron's own spiking nonlinearity (Fig. 2B). Importantly, this allows us to account for the rectification of a neuron's inputs imposed by the spike-generation process. The NIM can thus be viewed as a ‘second-order’ generalization of the LN model, or an LNLN cascade [59], [60]. Previous work from our lab [45] cast this model structure in a probabilistic form, and suggested several statistical innovations in order to fit the models using neural data [45], [61], [62]. Here, we present a general and detailed framework for NIM parameter estimation that greatly extends the applicability of the model. This model structure has also been suggested for applications outside of neuroscience in the form of projection pursuit regression [63], including generalizations to response variables with distributions from the exponential family [64].

The processing of the NIM is comprised of three stages (Fig. 2C): (a) the filters **k** *_i_* that define the stimulus selectivity of each input; (b) the static ‘upstream’ nonlinearities *f_i_*(.) and corresponding linear weights *w_i_* which determine how each input contributes to the overall response; and (c) the spiking nonlinearity *F*[.] applied to the linear sum over the neuron's inputs. The predicted firing rate *r*(*t*) is then given as:
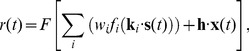
(1)where **s**(*t*) is the (vector-valued) stimulus at time *t*, **x**(*t*) represents any additional covariates (such as the neuron's own spike history), and **h** is a linear filter operating on **x**. Note that equation (1) reduces to a GLM when the *f_i_*(.) are linear functions. The *w_i_* can also be extended to include temporal convolution of the subunit contributions to model the time course of post-synaptic responses associated with individual inputs [45], as well as ‘spatial’ convolutions to account for multiple spatially distributed inputs with similar stimulus selectivity [65]. Since equivalent models can be produced by rescaling the *w_i_*, and *f_i_*(.) (see Methods), we constrain the subunit weights *w_i_* to be either +/−1. Because we generally assume the *f*_i_(.) are rectifying functions, the *w_i_* thus specify whether each subunit will have an ‘excitatory’ or ‘inhibitory’ influence on the neuron.

Parameter estimation for the NIM is based on maximum likelihood (or maximum a posteriori) methods similar to those used with the GLM [6]–[8]. Assuming that the neuron's spikes are described in discrete time by a conditionally inhomogeneous Poisson count process with rate function *r*(*t*), the log-likelihood (*LL*) of the model parameters given an observed set of spike counts *R*_obs_(*t*) is given (up to an overall constant) by:
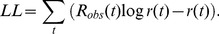
(2)


To find the set of parameters that maximize the likelihood (eq. 2), we adapt methods that allow for efficient parameter optimization of the GLM [7]. First, we use a parametric spiking nonlinearity given by *F*[*x*] = *α*log[1+exp(*β*(*x*-*θ*))], with scale *α*, shape *β*, and offset *θ*. Other functions can be used, so long as they satisfy conditions specified in [7]. This ensures that the likelihood surface will be concave with respect to linear parameters inside the spiking nonlinearity [7], and in practice will be well-behaved for other model parameters (see Fig. S1; Methods).

Because it is straightforward to estimate the linear term **h**, and the *w_i_* are constrained to be +/−1, the upstream nonlinearities *f_i_*(.) and the stimulus filters **k**_i_ are the key components that must be fit in the NIM. While it is typically not feasible to optimize the likelihood with respect to both sets of parameters simultaneously, an efficient strategy is to use block coordinate ascent [66], alternating between optimizing the **k** *_i_* and *f_i_*(.), in each case holding the remaining set of parameters constant (see Methods). ‘Linear’ parameters, such as **h** and *θ*, can be optimized simultaneously during either (or both) optimization stages.

While the set of ‘upstream nonlinearities’ *f_i_*(.) can be represented as parametric functions such as rectified-linear or quadratic functions (see Methods), a powerful approach is to represent them as a linear combination of basis functions *φ_j_*(.) such as piecewise linear “tent” basis functions, i.e., *f_i_*(*g*) = Σ*_j_ a_ij_φ_j_*(*g*) [17], [45]. In doing so, estimation of the upstream nonlinearities reduces to estimating linear parameters *a_ij_* inside the spiking nonlinearity, with a single global optimum of the likelihood function for a given set of stimulus filters **k**_i_.

For a fixed set of upstream nonlinearities, the stimulus filters **k**_i_ can be similarly optimized, although the resulting likelihood surface will not in general be convex because the **k**_i_ operate inside the upstream nonlinearities. Nevertheless, we have found that in practice their optimization is well-behaved and that local minima can be avoided with appropriate optimization procedures (Fig. S1; see Methods). Furthermore, it is straightforward to evaluate the likelihood function and its gradient with respect to the **k**_i_ analytically (see Methods), allowing for efficient gradient-based optimization.

Thus, optimal parameter estimates for the NIM can be determined efficiently, even for models with large numbers of parameters (see examples below). The time required for filter estimation (typically the most time-consuming step) scales approximately linearly with the experiment duration, the dimensionality of the stimulus, and the number of model subunits (Fig. S2). This is very favorable compared with alternative nonlinear modeling approaches such as MID [14], which require using simulated annealing and quickly becomes intractable as the number of filters and/or stimulus dimensions is increased.

Furthermore, because the NIM provides an explicit probabilistic model for the neuronal spike response, regularization of the model components can be incorporated without adversely affecting the behavior of the optimization problem [7] (see Methods). This is particularly important when optimizing high-dimensional spatiotemporal filters and/or models with many inputs, which are both discussed further below. Likewise, as with other probabilistic modeling approaches – but not those relying on spike-triggered measurements [67] – the model can be optimized using data recorded with natural stimulus ensembles (containing complex correlation structure, and non-Gaussian distributions) without introducing biases into the parameter estimates.

The NIM thus provides a nonlinear modeling framework in which large numbers of parameters can be efficiently estimated using data recorded with arbitrarily complex stimulus ensembles. In addition to this flexibility, the NIM provides model fits that are more directly interpretable due to its physiologically motivated model structure. To illustrate these advantages, below we first apply the NIM to the example ON-OFF RGC from Fig. 1, and then demonstrate its wide applicability on recorded and simulated neurons in several different sensory areas.

### Nonlinear models of the ON-OFF retinal ganglion cell example

**Figure 3:**
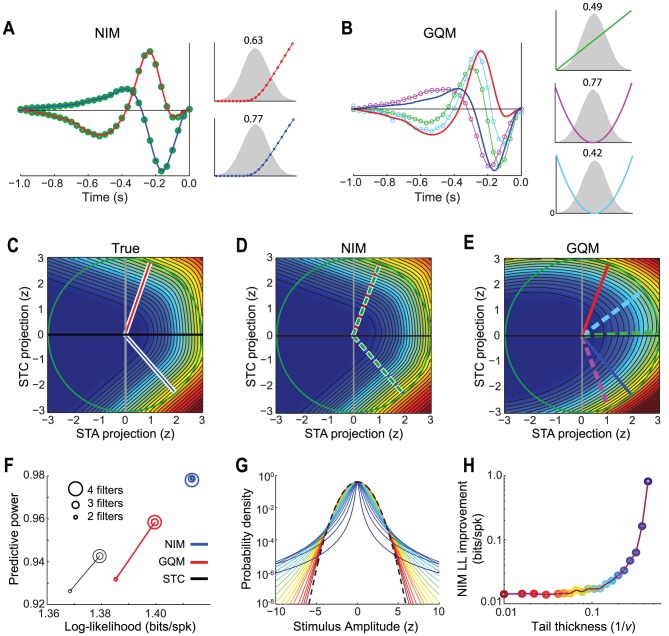
Comparison of NIM and quadratic model. **A**) The filters fit by the NIM (green dots) are able to capture the true underlying ON and OFF filters (red and blue), as well as the shape of the upstream nonlinearities (right), which are shown relative to the corresponding distributions of the filtered stimulus (gray shaded). The ranges of the y-axes for different subunits are indicated by the numbers above, for comparison of their relative magnitudes. These ‘subunit weights’ are scaled so that their squared magnitude is one. **B**) The filters fit by the GQM, consisting of two (excitatory) squared filters (magenta and light blue) and a linear filter (green trace), are different than the true filters (red and blue), but are in the same subspace, as demonstrated in (E). **C**) The simulated neuron's response function (shaded color depicts firing rate) and true filters (red and blue) projected into the STC subspace (identical to Fig. 1F). **D**) Response function predicted by the NIM. The filters identified by the NIM (dashed green) overlay onto the true filters. **E**) Same as (D) for the GQM, with colored lines corresponding to the filters in (B). **F**) Model performance is plotted for the STC, GQM, and NIM fit with different numbers of filters (indicated by different circle sizes). Log-likelihood (relative to the null model) is shown on the x-axis, and the ‘predictive power’ [99] is shown on the y-axis; both were evaluated on a simulated cross-validation data set. The NIM (blue) outperforms the GQM (red), both of which outperform a nonlinear model based on the STC filters (black, see Methods). The STC model and GQM achieve maximal performance with 3 filters, since this is sufficient for capturing the best-fit quadratic function in the relevant 2-D stimulus subspace, while the NIM achieves optimal performance with two filters, as expected. **G**) To determine how model performance depends on the stimulus distribution we simulated the same neuron's response to white noise luminance stimuli with Student's *t*-distributions, ranging from Gaussian (i.e., *ν* = ∞, dashed black) to “heavy tailed” (decreasing *ν* from red to blue). **H**) The log-likelihood improvement of the NIM over the GQM increases as a function of the tail thickness (parameterized by 1/*ν*) of the stimulus distribution (which also determines the tail thickness of the filtered stimulus distributions). The GQM is able to provide a very close approximation for large values of *v* (i.e., a more normally-distributed stimulus), but has lower performance compared to the NIM for more heavy-tailed stimuli.

Returning to the example ON-OFF RGC (Fig. 1), the NIM is a natural choice given that its structure matches that of the simulated neuron. Using the estimation procedures described above, the NIM is able to successfully capture the true stimulus selectivity of its individual inputs (Fig. 3A), including the ‘upstream nonlinearities’ associated with each input, as well as the form of the spiking nonlinearity (see Methods).

This example thus illustrates the core motivation behind the NIM of modeling a neuron's stimulus processing in terms of rectified neuronal inputs. While the structure of the simulated RGC neuron in this example may appear to be a convenient choice, its form is consistent with other models of ON-OFF processing [48], [53], and with models of RGCs in general [54], [68].

Thus, to understand the advantages and disadvantages of the NIM structure, it is useful to compare it with the dominant alternative approach for describing nonlinear stimulus processing: “quadratic models”. Such models have recently been cast in an information-theoretic context [15], [27], [28], as well as in the form of an explicit probabilistic model [26] which has been referred to as the ‘Generalized Quadratic Model’ (GQM). The GQM can be viewed as a probabilistic generalization of STA/STC analysis [26] and of the second-order Wiener-Volterra expansion [20]. The GQM can also be written in the form of a NIM where the upstream nonlinearities *f_i_*(.) are fixed to be linear or squared functions:
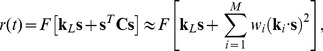
(3)where **k** *_L_* is a linear filter, and the *M* squared filters **k** *_i_* generally provide a low-rank approximation to the quadratic component **C** [26]. In this sense, the probabilistic framework described here is easily extended to encompass quadratic models, providing a means for direct comparison between different nonlinear structures.

For the ON-OFF RGC, the GQM finds one linear and two quadratic filters, all of which are contained in the two-dimensional subspace identified by STC analysis, meaning that the GQM filters are also linear combinations of the true ON and OFF filters (Fig. 3B). Note that while two filters are sufficient to span the relevant stimulus subspace, the third GQM filter provides an additional degree of freedom to capture the best quadratic approximation to the underlying ‘neural response function’ (Fig. 3E).

Although in this example the resulting quadratic function cannot completely capture the form of the response function constructed from rectified inputs, we note that it still provides a good approximation, as shown by only modest reductions in model performance compared to the NIM (Fig. 3F). However, as expected from a second-order Taylor series expansion, such an approximation breaks down further from the “origin” of the subspace. Thus, the quadratic approximation will typically be less robust for stimuli with heavy-tailed distributions such as those associated with natural stimuli [69]–[71]. To illustrate this point we performed simulations of the same ON-OFF RGC presented with white noise stimuli having a Student's *t*-distribution, where the tail thickness was controlled by varying the number of degrees of freedom (Fig. 3G). The improved performance of the NIM over the GQM is indeed substantially enhanced for stimulus distributions with heavier tails (Fig. 3H). We also verified similar effects for a range of simulated neurons (data not shown).

We emphasize that one of the key advantages of the NIM over previously described methods is that it provides a more interpretable picture of stimulus processing as a sum of rectified neuronal inputs. As we demonstrate through several examples below in both the visual and auditory systems, it appears that sensory computation by neurons will often adhere to this general form, which is motivated primarily by physiological, rather than mathematical, considerations.

### Inferring the interplay of excitation and inhibition in visual and auditory neurons

One of the main advantages of the NIM structure is the ability to specifically model the effects of inhibitory inputs, which are increasingly being shown to have a large impact on neuronal processing in many sensory areas [72]–[74]. Indeed, the NIM generates predictions of the functional tuning of excitation and inhibition, and provides insight into their role in sensory processing. To demonstrate this, we apply the NIM to example neurons from visual and auditory areas.

**Figure 4:**
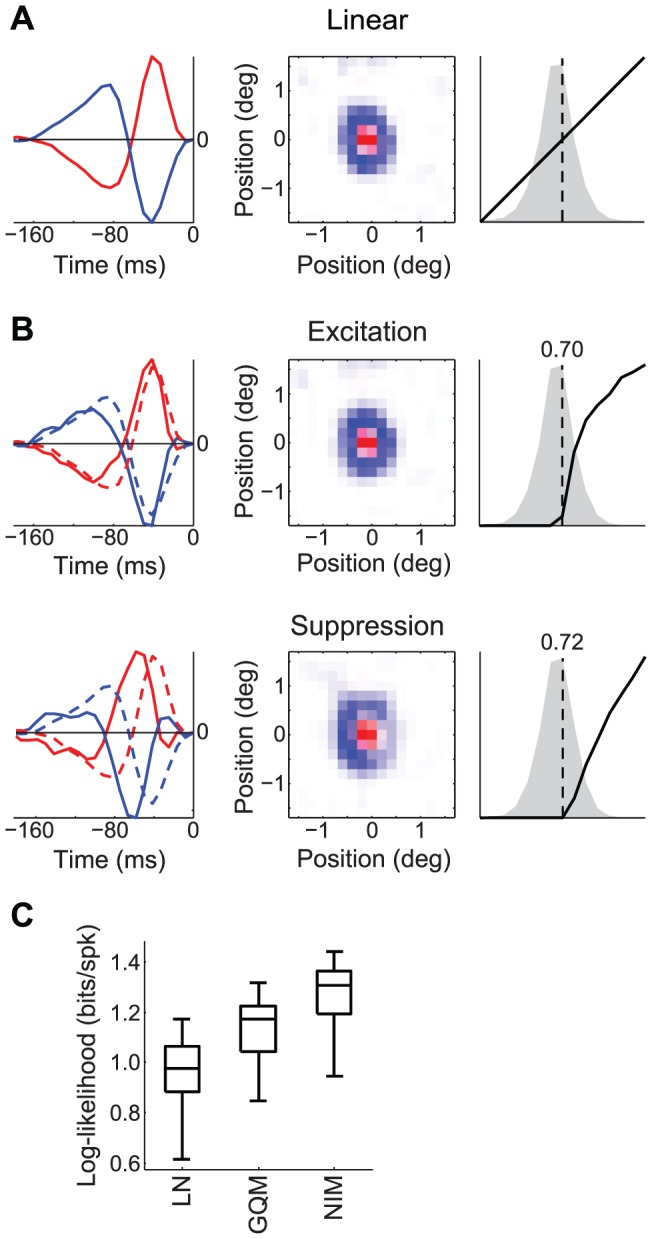
Spatiotemporal tuning of excitatory and suppressive inputs to an LGN neuron. A) The linear receptive field can be represented as the sum of two space-time separable components, corresponding to the receptive field center (red) and surround (blue). B) The NIM with excitatory (top) and suppressive (i.e., putative inhibitory, bottom) inputs. The excitatory and suppressive components (solid) both have slower, and less biphasic, temporal responses (left) compared with the linear model (dashed). The suppressive input is also delayed relative to the excitatory input. Both excitatory and suppressive inputs have roughly the same spatial profiles (middle), and both provide rectified input through the corresponding upstream nonlinearities (right). C) The NIM has significantly better performance, as measured by cross-validated log-likelihood, compared to the linear model (*p* = 0.002; *n* = 10 cross-validation sets; Wilcoxon signed rank test) and the GQM (*p* = 0.002).

We first consider an example cat LGN neuron during the presentation of natural movies [45], [75], [76]. Accurate characterization of LGN processing poses substantial challenges for previous nonlinear approaches, due to the high temporal resolution of LGN responses in this context [77] combined with the large number of spatial dimensions of the stimulus. As a result, previous nonlinear applications have either utilized lower temporal resolutions [78], [79] or parametric models of the spatial processing [38], [45], [80]. The methods described here allow for (appropriately regularized) spatiotemporal receptive fields (STRFs) of LGN neurons to be fit at sufficiently high resolution, using natural movies. We find that the response of the example LGN neuron consists of an excitatory receptive field that is delayed relative to the linear STRF (Fig. 4A), along with a second, more delayed ‘suppressive’ receptive field (Fig. 4B), corresponding to putative inhibitory input. Unlike in previous studies, the tractability of the fitting procedures used here allows for high spatial and temporal resolution of the putative inputs (Fig. 4B), as well as the application of sparseness and smoothness regularization (see Methods). By comparison, the GQM identifies similar STRFs, but has worse performance (Fig. 4C), as well as a different nonlinear structure and resulting physiological interpretation (Fig. S3).

**Figure 5:**
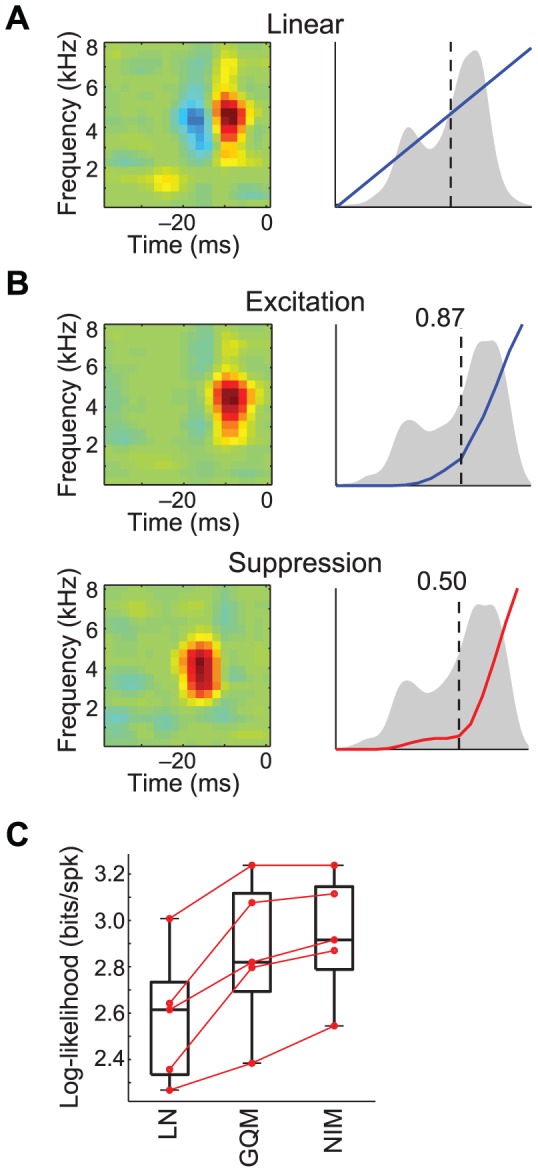
Spectrotemporal tuning of excitation and suppression in the songbird auditory midbrain. A) The linear spectrotemporal receptive field (STRF; left) contains two subfields of opposite sign. B) The excitatory (top) and suppressive (bottom) spectrotemporal filters identified by the NIM are similar to the positive and negative subfields of the linear STRF respectively. However, these inputs are both rectified by the upstream nonlinearities (right), resulting in different stimulus processing (see Fig. S4). C) Comparison of log-likelihoods of the LN model, GQM, and NIM. Red lines show the performance across models for each cross-validation set. Note that the duration of the recording, and the neuron's relatively low firing rate, limit the statistical power of model comparisons.

Next we consider an example neuron from zebra finch area MLd, as the animal is presented with conspecific bird songs [81]–[83]. These neurons respond to specific frequencies of the song input, and hence their stimulus selectivity can be characterized by a linear spectrotemporal receptive field (STRF) [9], which can be recovered in an unbiased manner using maximum-likelihood estimation (Fig. 5A) [84] despite the presence of higher order correlations in the stimulus. Application of the NIM to this example neuron again recovers both an excitatory and a temporally delayed suppressive component (Fig. 5B). The description of the neuron's stimulus tuning provided by the NIM is closely related to that given by the linear model, but instead of identifying positive and negative domains of the linear STRF as excitatory and suppressive, these effects are segregated into different nonlinear processing subunits, each individually rectified. The separate excitatory and suppressive inputs provide a more accurate description of the underlying stimulus processing than a single linear STRF, as demonstrated by the significantly improved model performance of the NIM compared with the LN model (Fig. 5C). As with the LGN example, the GQM identifies similar excitatory and suppressive filters as the NIM, but again provides a less physiologically interpretable description of the underlying computation (Fig. S4), and has comparable, if slightly reduced, performance (Fig. 5C).

### Modeling complex neural response functions in terms of rectified inputs

Thus far we have only considered cases where the neuron's response is described by a NIM with a small number of inputs, consistent with simpler stimulus processing in sub-cortical areas. In contrast, in the visual cortex, even V1 ‘simple cells’ can exhibit selectivity to large numbers of stimulus dimensions [57], [62]. Further, the dominant model of V1 ‘complex cells’ is the nonlinear “Energy Model” [10], [85]–[87], which posits quadratic stimulus processing that results in the response representing the amount of local, oriented, band-pass “stimulus energy”. The Energy Model has been broadly tested [10], [87], and is well supported by previous nonlinear modeling approaches [13], [26], [27], [57], [88]. While the Energy Model provides a functional description of stimulus processing for V1 complex cells, it is less clear how such stimulus selectivity is constructed, and how it is related to V1 simple cell processing. Here we demonstrate that the NIM can describe both simple and complex cell processing as a sum of rectified inputs, providing a basis for a unified description of visual cortical neuron computation [62].

**Figure 6:**
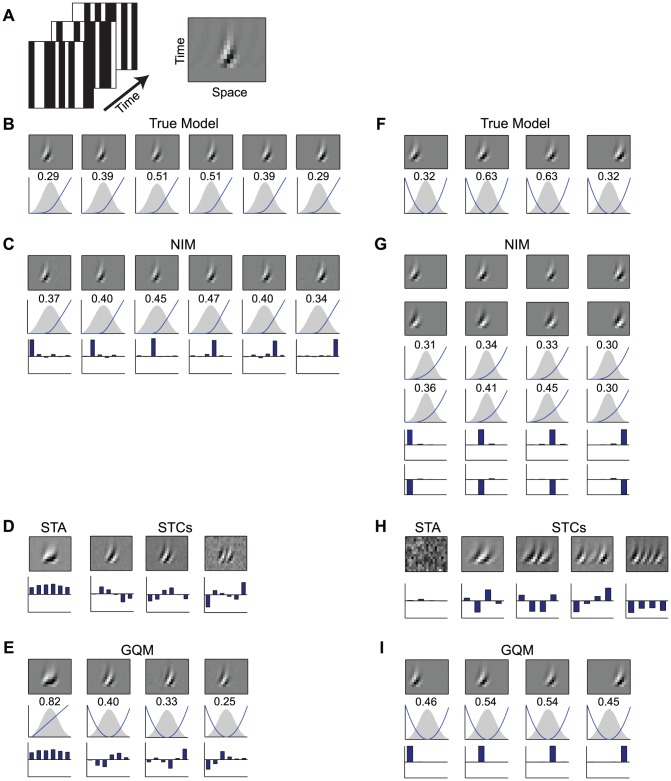
Modeling stimulus selectivity arising from many inputs. A) Simulated V1 neurons are presented with one-dimensional spatiotemporal white noise stimuli (left). Their stimulus processing is constructed from a set of spatiotemporal filters (example shown at right), depicted with one spatial dimension (x-axis) and time lag (y-axis). B) The first simulated neuron is constructed from six spatially overlapping direction-selective filters (top), similar to those observed experimentally for V1 neurons. Below, the corresponding filtered stimulus distributions are shown along with the respective upstream nonlinearities (blue). C) The NIM identifies the correct spatiotemporal filters (top), as well as the form of the upstream nonlinearities (middle). The projections of the NIM filters onto the true filters (bottom) illustrate that the NIM identifies the true filters. D) The STA for the simulated neuron (left), along with the three significant STC filters (right) are largely contained in the subspace spanned by the true filters, but reflect non-trivial linear combinations of these filters (bottom). E) The GQM is composed of a linear input (left) and three excitatory squared inputs (right). While the GQM filters are more similar to the true filters, they also represent non-trivial linear combinations of them (bottom). F) The second simulated neuron consists of four similar, but spatially shifted, inputs that are squared. G) The NIM represents each true (squared) input by an opposing pair of rectified inputs. H) The STA (left) does not show any structure because the neuron's response is, by construction, symmetric in the stimulus. The four significant STC filters (right) represent distributed linear combinations of the four underlying filters. I) The GQM recovers the correct stimulus filters, given appropriate sparseness regularization.

We first consider two simulated V1 neurons in order to demonstrate the capacity for such a unified description, before applying the NIM to experimental data. We generate simulated data using a one-dimensional white-noise bar stimulus aligned with the simulated neurons' preferred spatial orientation (Fig. 6A), which is a common, relatively low-dimensional, stimulus used in nonlinear characterizations of V1 neurons [57], [88], [89]. The first simulated neuron's response is constructed as a sum of six rectified direction-selective inputs (Fig. 6B), consistent with the structure of the NIM, while the second neuron's response is constructed from four such inputs processed by a squaring nonlinearity, similar to the standard Energy Model of V1 complex cells [85].

For the neuron with rectified inputs, the NIM fitting procedure is indeed able to identify the true underlying stimulus filters and the form of the rectifying upstream nonlinearities (Fig. 6C). Additionally, while the optimal number of filters can be determined using the cross-validated model performance, the identified stimulus filters, and the resulting model performance itself, are relatively insensitive to specification of the precise number of model subunits (Fig. S5). This demonstrates the ability of the NIM to robustly identify even relatively complex stimulus processing, in cases where such processing arises from a sum of rectified inputs.

Furthermore, as with the ON-OFF RGC example above (Figs. 1 and 3), STC analysis of this simulated V1 neuron can identify the appropriate stimulus subspace, although not the true underlying filters (Fig. 6D). Because of the high dimensionality of the resulting subspace, however, it is more difficult to estimate the mapping from the subspace to the neuronal response compared with the ON-OFF example. The lack of alignment between the STA/STC filters and the true filters further complicates a straightforward interpretation of the estimated function.

By comparison, the GQM identifies filters with characteristics that more closely resemble those of the true input filters (e.g., more localized, fewer lobes). The improved performance of the GQM compared with an STC-based model (Fig. 6E) highlights the greater power and flexibility of a probabilistic modeling framework, particularly the importance of regularization. Nevertheless, the GQM filters still reflect non-trivial linear combinations of the true filters, as with the STC filters (Fig. 6E, *bottom*).

Of course, one would expect the NIM to outperform other models when the generative model is composed as a sum of rectified inputs. In a second simulated example, however, we illustrate the flexibility of the NIM in capturing other neural response functions. The second simulated neuron is constructed from four direction-selective inputs that are squared and summed together to generate a quadratic response function (Fig. 6F). The NIM is still able to identify the true generative model using pairs of rectified inputs with equal but opposite input filters to represent each quadratic filter (Fig. 6G). This representation is certainly not the most efficient in this case, as the GQM is able to identify the correct filters (Fig. 6I) using fewer parameters and a more straightforward estimation procedure.

These two simulated V1 examples thus illustrate the potential tradeoffs between the NIM and GQM. On the one hand, the NIM provides a more flexible framework that can capture a broader range of nonlinear stimulus processing. In fact, any response function can in principle be represented with this structure [90]. The NIM structure is also more appropriate for explicitly modeling neuronal inputs, and thus allows for more plausible physiological interpretation of its components. On the other hand, the GQM can capture the nonlinear mapping up to second order more efficiently, and identifies the relevant stimulus subspace robustly. This suggests the potential for combining these approaches when investigating complex neuronal processing, such as by using the GQM to identify the relevant stimulus subspace and provide initial estimates of the number and properties of NIM filters, followed by application of the NIM framework (see Methods; Figs. S1, S3).

### Application of the NIM to recorded V1 neurons

**Figure 7:**
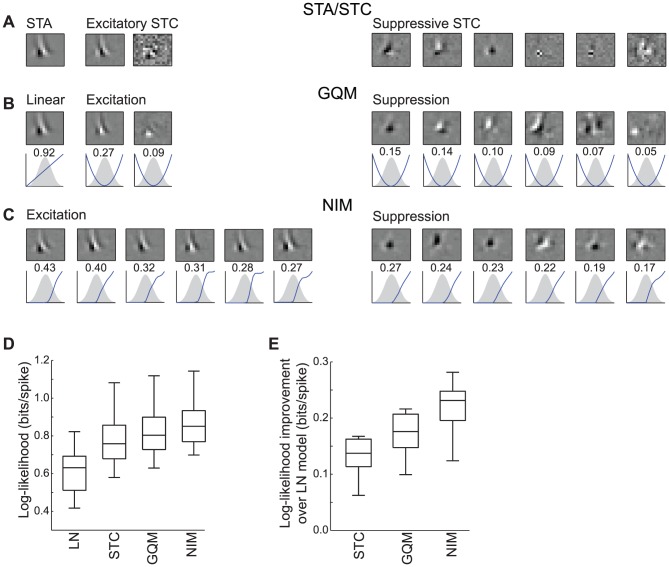
Models of multi-input stimulus processing in a V1 neuron. A) Standard spike-triggered characterization for this neuron reveals a ‘complicated simple-cell’ response [62], with a clear direction-selective STA (left), two excitatory STC filters (middle), and six suppressive STC filters (right). B) The GQM identifies a set of filters (one linear, two squared excitatory, and six squared suppressive) that are roughly similar to the STA/STC filters, but with smoother and sparser spatiotemporal structure (due to regularization). C) The NIM filters (top) and upstream nonlinearities (bottom) reveal a similar description of the stimulus processing, although with greater consistency among the (six) excitatory and (six) suppressive stimulus filters. D) Comparison of the cross-validated log-likelihood of the LN model (one linear filter), the ‘STC model’ given by fitting a GLM to the outputs of the STA/STC filters (see Methods), the GQM, and the NIM. Given the neuron's simple-cell-like response (i.e., large weight of the STA), a large fraction of the response can be captured with the linear filter alone (the LN model). Nevertheless, all three multi-filter models provide substantial improvements compared to the LN model. E) In order to compare the performance of the nonlinear (multi-filter) models directly, their improvement relative to the LN model is depicted. This shows that the GQM significantly outperforms the ‘STC’ model (*p* = 0.002; *n* = 10; Wilcoxon signed rank test), and that the NIM similarly outperforms the GQM (*p* = 0.002).

While the simulated examples above allowed for model comparisons when the neurons' response functions were known, they also provide a foundation for understanding model fits to real V1 data. We first consider a V1 neuron recorded from an anesthetized macaque in the context of similar one-dimensional white noise stimuli [57]. While this neuron has a clear STA and is considered a simple cell by classical measures, STC analysis identifies two excitatory and six suppressive stimulus dimensions (based on inspection of the eigenvalue spectrum) in addition to the STA (Fig. 7A). In this case, the GQM identifies similar filters to STC analysis, although the application of smoothness and sparseness regularization allows it to resolve more realistic stimulus filters (Fig. 7B), and produce significantly improved model performance (Fig. 7D) compared to an STC-based model (see Methods).

We also fit a NIM with six excitatory and six suppressive stimulus filters, where the number of filters was selected based on cross-validated model performance (Fig. 7C; see also Fig. S5). As expected, these 12 filters span a stimulus subspace that is largely overlapping with the subspace identified by the GQM. However, the additional stimulus filters, and the inferred upstream nonlinearities associated with each subunit, allow the NIM to capture additional aspects of the neural response function that significantly improve the cross-validated model performance relative to the quadratic models (Figs. 7D, E). We also note that the NIM appears to identify a more consistent set of stimulus filters than the quadratic models.

**Figure 8:**
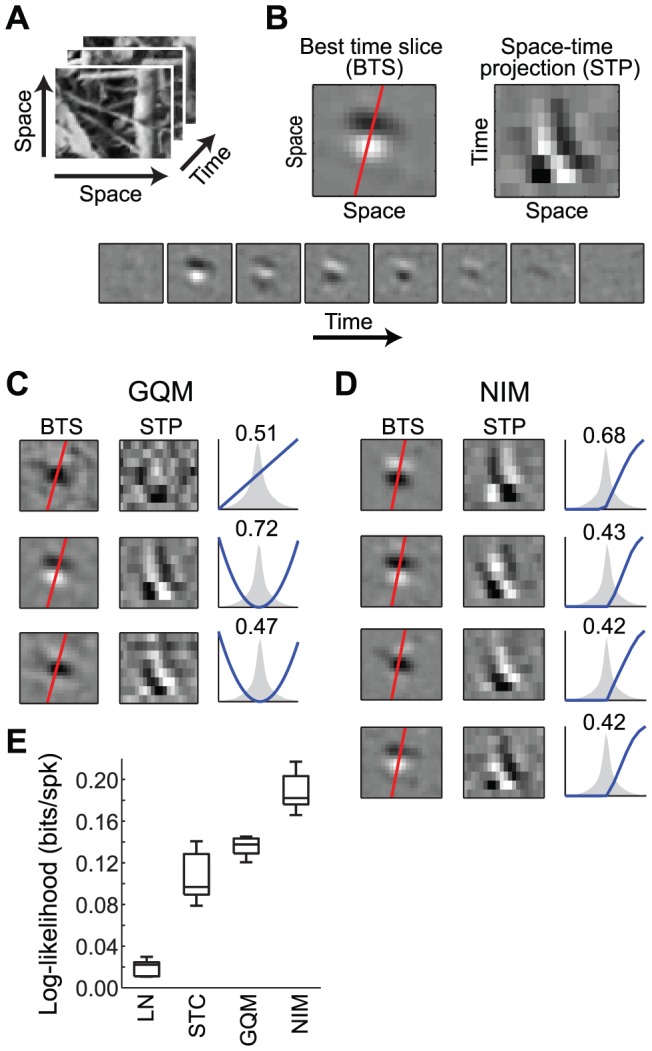
Models of a V1 neuron in the context of natural stimuli. A) The natural movie stimulus used here has two spatial and one temporal dimension. B) The neuron's response is characterized in terms of three-dimensional spatiotemporal filters. An example spatiotemporal filter is comprised of a spatial filter at each time step (at 20 ms resolution). To simplify the depiction of each filter, we take advantage of their stereotyped structure, and plot the spatial distribution at the best time slice (BTS, left), as well as the space-time projection (STP, right) along an axis orthogonal to the preferred orientation (red line; see Methods). C) The GQM for this neuron consists of one linear (top) and two excitatory squared filters (bottom). The BTS and STP for each filter are shown at left, and the distributions of the filtered stimulus, and associated nonlinearities, are shown at right. Note that the two squared filters roughly form a ‘quadrature pair’ of direction-selective Gabor filters. There is also a linear filter (top), which has less clear spatial structure, and is not direction-selective. D) The NIM consists of four excitatory filters (left) that are qualitatively similar to the quadrature pair of GQM filters. However, by identifying four inputs with inferred upstream nonlinearities (right), the NIM has greater flexibility in describing the neuron's computation. E) Comparison of model performance for the LN and STC-based models, as well as the GQM and NIM, showing that the NIM substantially outperformed other models for this neuron.

Similar comparisons also come to light in when applying the models to V1 complex cells, even in the most demanding stimulus contexts. To illustrate this, we consider an example V1 neuron recorded from an anesthetized cat presented with natural and naturalistic stimuli (Fig. 8A) [62], [91]. Because the stimuli are sequences of two-dimensional images, the required spatiotemporal stimulus filters span two dimensions of space and one dimension of time (Fig. 8B), resulting in a very large number of parameters associated with each subunit. Nevertheless, the parameters of the GQM and NIM can be estimated directly utilizing appropriate regularization (see Methods).

The GQM estimated for this neuron is comprised of a pair of excitatory, direction-selective squared filters, as well as a weaker, non-direction-selective linear filter (Fig. 8C). This characterization reflects the neuron's spatial-phase invariance, and is thus consistent with an Energy Model description. While such selectivity suggests that this neuron would be ideally suited for a quadratic model, the NIM (Fig. 8D) significantly outperforms both the GQM and a whitened STC-based model [9], [58], [62] (Fig. 8E).

The NIM identifies four rectified excitatory inputs that share similar spatial tuning and direction selectivity, but with different spatial phases (Fig. 8D). This description is similar to that provided by the quadratic terms of the GQM, but by identifying the nonlinearities associated with each of these inputs individually, the NIM has additional flexibility that results in improved performance (Fig. 8E). This suggests that a description of complex cells using physiologically plausible inputs (in the form of the NIM) may be a viable alternative to the Energy Model. The improved performance of the NIM is also likely due, at least in part, to the heavy-tailed distribution associated with the naturalistic movie stimuli (as described above, Figs. 3G, H).

Thus, the application of the NIM to V1 neurons further illustrates the generality of the method, and specifically emphasizes its ability to capture substantially more complex stimulus processing, with large numbers of inputs. We note that because cortical neurons are several synapses removed from receptor neurons, a cascade model with a longer chain of upstream LN components might be more appropriate, although existing methods could not be used for parameter estimation with such a model. The ability of the NIM to capture a given neuron's stimulus processing thus relates to the extent to which the upstream neurons themselves can be approximated by LN models. In cases where this assumption is not appropriate, one can apply a fixed nonlinear transformation to the stimulus resembling the response properties of upstream neurons [31], [32], thus allowing the problem to be cast into a more general NIM framework.

### Conclusions

We have presented a physiologically inspired modeling framework, the NIM, which extends several recently developed probabilistic modeling approaches. Specifically, the NIM assumes a form analogous to an integrate-and-fire neuron, whereby a neuron receives a set of rectified excitatory and inhibitory inputs, each of which is assumed to process the stimulus linearly. The parameters can be estimated robustly and efficiently, and the resulting model structure is able to capture a broader range of neural responses than previously proposed probabilistic methods. Importantly, the physiologically inspired model structure of the NIM also allows for greater interpretability of the model fits, as the components of the model take the form of stimulus-driven excitatory and inhibitory inputs. The NIM thus provides a framework for connecting nonlinear models of sensory processing directly with the underlying physiology that can be applied in a range of sensory areas and experimental conditions.

## Methods

### Parameter estimation details

As described above, the key parameters in the NIM are the stimulus filters **k**_i_ and the set of coefficients *a*_ij_ representing the upstream nonlinearities *f*_i_(.). While these parameters cannot generally be optimized simultaneously, a powerful approach is to use block coordinate ascent [66] and alternate between optimizing the filters **k**_i_, and upstream nonlinearities *f*_i_(.), holding the remaining parameters fixed in each iteration. The parameters of the spiking nonlinearity function *F*[*x*; *α*,*β*,*θ*] = *α*log[1+exp(*β*(*x*-*θ*))] can also be estimated iteratively, or as a final stage after convergence of the **k**_i_ and *f*_i_(.) (which we find is typically sufficient). Note that the parameter *β* is not generally identifiable in the model (being degenerate with the coefficients *a_ij_* of the upstream nonlinearities), but joint estimation of *α* and *β* after the other model parameters are fixed allows for a more precise final fit to the spiking nonlinearity function.

Thus, at each stage of the fitting procedure we have the problem of maximizing a (penalized) log-likelihood function with respect to some subset of parameters, while holding a remaining set of parameters fixed. In all cases, we use a standard line search strategy to locate an optimum of the likelihood function given some initial values for the parameters. Because we are often optimizing very high-dimensional parameter vectors (specifically when optimizing the **k**_i_), we use a quasi-Newton method with a limited-memory BFGS approximation of the inverse Hessian matrix [92] to determine the search direction. This code is implemented in the *Matlab* function “minFunc”, provided by Mark Schmidt (available at http://www.di.ens.fr/~mschmidt/). When using sparseness (L1) regularization we utilize the *Matlab* package “L1General”, also provided by Mark Schmidt. When optimizing the coefficients *a*_ij_ of the upstream nonlinearities we additionally enforce a set of linear constraints (described below), and in such cases we utilize *Matlab*'s constrained optimization routine “fmincon”. A *Matlab* implementation of the NIM parameter estimation routines described here is available from our website: (http://www.clfs.umd.edu/biology/ntlab/NIM/)

### Optimizing the filters k_i_

Optimization of the filters can be accomplished efficiently by analytic calculation of the log-likelihood gradient with respect to the **k**_i_, which is given by:
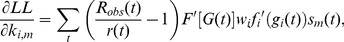
(4)where the ‘internal generating function’ 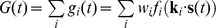, *F*'[.] and *f_i_*'(.) are the derivatives of *F*[.] and *f_i_*(.) with respect to their arguments, and *s*_m_(t) is the *m* ^th^ element of the stimulus at time *t*. While the likelihood surface is not generally convex with respect to the **k**_i_, the optimization problem is well-behaved in practice. We note that while the derivatives of the *f_i_*(.) are discontinuous (piece-wise constant) when using the tent-basis representation (eq. 6 below), gradient-based optimization methods still provide robust results, in particular because we use regularization to enforce smooth *f_i_*(.) such that the contribution of the discontinuities to the overall log-likelihood gradient is negligible in practice.

To diagnose the presence of undesirable local maxima, and to identify the global optimum of the likelihood function, we use repeated random initializations of our optimization routine (Fig. S1). In some cases, such as the ON-OFF RGC example (Figs. 1, 3), this approach reveals that the choice of initial values for **k**_i_ does not affect the identified local optimum. In other cases, the likelihood surface will contain more than one distinct local maximum, although usually only a small number. For example, when optimizing the filters for the example MLd neuron (Fig. 5) we found two distinct local optima of the likelihood function. For models with large numbers of subunits, the filter optimization remains well-behaved, generally identifying a relatively small number of local optima that correspond to similar models (Fig. S1).

This procedure can be greatly sped up by initially optimizing the filters in a low-dimensional stimulus subspace, rather than in the full stimulus space. Such subspace optimization has been previous used in conjunction with STC analysis to identify the relevant stimulus subspace [15], [62], [93]; however the GQM provides a means of generalizing the robust subspace identification properties of STC analysis to arbitrary non-Gaussian stimuli, and in cases where regularization is important. With a low-dimensional subspace identified the filters of a NIM can be rapidly optimized, and many filter initializations can be tested.

### Fitting the upstream nonlinearities *f*_i_(.)

We begin the NIM fitting with its upstream nonlinearities *f*_i_(.) initialized to be threshold-linear functions:
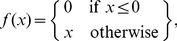
(5)and perform initial estimation of the filters. While other rectifying functions can be used, the use of scale-invariant functions such as this one has the advantage that the effect of the upstream nonlinearity is independent of the scale of the filter.

After estimating the **k**_i_, we then estimate the *f*_i_(.) nonparametrically, as a linear combination of a set of piecewise linear basis functions *f_i_*(*g*) = Σ*_j_ a_ij_φ_j_*(*g*) [17], [45], while holding the **k**_i_ fixed. These basis functions are given by:
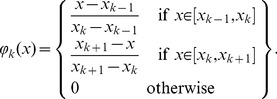
(6)These piecewise linear functions are particularly useful as they provide a set of localized basis functions, requiring only that we choose a set of ‘grid points’ *x*_k_. These points can be selected by referencing the distribution of the argument of *f*_i_(.), i.e., *p*(*g_i_*) where *g*_i_(t) = **k**_i_•**s**(*t*), either at *n*-quantiles of *p*(*g_i_*), or at uniformly spaced points across the support of *p*(*g_i_*). In order to encourage interpretability of the model subunits as ‘neural inputs’, we constrain the *f*_i_(.) to be monotonically increasing functions by using a system of linear constraints on the *a*_ij_ during optimization. Because the model is invariant to shifts in the ‘y-offset’ of the *f*_i_(.) (which can be absorbed into the spiking nonlinearity function), we add the additional set of constraints that *f*_i_(0) = 0 to eliminate this degeneracy. Furthermore, changes in the upstream nonlinearities can influence the effective regularization of the **k**_i_, by altering how each **k**_i_ contributes to the model prediction. As a result, the coefficients *a*_ij_ are rescaled after each iteration so that the standard deviation of each subunit's output is conserved. This ensures that the upstream nonlinearities do not absorb the scale of the **k**_i_.

### Regularization

An important advantage of explicit probabilistic models such as the NIM is the ability to incorporate prior knowledge about the parameters via regularization. Because each of the filters **k**_i_ often contains a large number of parameters, regularization of the filters is of particular importance, as discussed elsewhere in the context of the GLM [9], [58], [84], [94]–[97], as well as other nonlinear models [26]. Such regularization can impose prior knowledge about the smoothness [9], [26], [94], sparseness [58], [84], [94]–[96], and localization [62], [97] of filters in space, frequency and time.

We consider several different forms of regularization in the examples shown, to encourage the detection of smooth filters with sparse coefficients. Specifically, we add a general penalty term of the form:
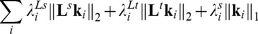
(7)to the equation for the log-likelihood (equation 2), where **L^s^** and **L^t^** are the discrete Laplacian operators with respect to spatial (or spectral) and temporal dimensions respectively, and *λ_i_^Ls^* , *λ_i_^Lt^* and *λ_i_^s^* are hyperparameters which determine the strength of spatial and temporal smoothness, and sparseness regularization, respectively. Other types of regularization, such as those that encourage localized filters [62], [97], as well as approximate Bayesian techniques for inferring hyperparameters [26], [58], [94], [96] could be incorporated as well, although we do not do so here.

Because we also expect the upstream nonlinearities *f*_i_(.) to be smooth functions, we incorporate penalty terms when estimating the parameters of the *f*_i_(.). Because we represent the *f*_i_(.) as linear combinations of localized tent basis functions: *f*_i_(.) = *a*_ij_ *φ*_j_(.), we can encourage smooth *f*_i_(.) by applying a penalty of the form: *λ*_i_^L^∥**L** *a*_ij_∥_2_ to the set of coefficients *a*_ij_ corresponding to a given *f*_i_(.), where **L** is again the one-dimensional discrete Laplacian operator.

In general, the hyperparameters can be inferred from the data using Bayesian techniques [94], or estimated using a (separate) cross-validation data set. Both methods can be time-consuming, however, and in practice we find that similar results can be achieved by ‘manually’ tuning the hyperparameters to produce filters **k**_i_ and upstream nonlinearities *f*_i_(.) with the expected degree of smoothness/sparseness. To demonstrate that our results were not overly sensitive to the selection of hyperparameters, we compare the NIM and GQM fit to the example V1 neuron from Fig. 8 using a range of regularization strengths (Fig. S6).

### Evaluating model performance

To evaluate model performance, we use *k*-fold cross-validation, in general taking the log-likelihood as a performance metric. The likelihood has the advantage over related measures such as *R^2^* in that it does not require repeated stimulus presentations to estimate, and thus can be applied to most data sets. It can also capture goodness-of-fit when spike history terms are incorporated [35]. Subtracting the log-likelihood of the null model (that predicts a constant firing rate, independent of the stimulus) provides a measure of the information carried by the spike train about the stimulus, in units of bits per spike [45], [98]. This measure is also directly related to the more traditional measure of deviance, which compares the log-likelihood of the estimated model to that of the ‘saturated’ model. In order to provide a more direct connection to standard measures of model performance based on repeated presentations of a stimulus, we also computed the ‘predictive power’ of the models for the simulated ON-OFF RGC (Fig. 3F), which is defined as the fraction of ‘explainable’ variance accounted for by the model [99]. Due to the lack of sufficient repeat trial data for our recorded data examples we could not compute this measure in those cases, however qualitatively similar results would be expected.

### Model selection

While selection of the optimal number of excitatory and suppressive subunits can be performed using standard model selection techniques, such as nested cross-validation, this choice can also often be guided by the specific application. Importantly, we find that the subunits identified by the NIM, as well as its performance, are generally robust towards precise specification of the number of excitatory and suppressive subunits, with ‘nearby’ models typically providing a very similar characterization of the neurons' stimulus processing (Fig. S5). This robustness is further aided by the incorporation of sparseness regularization on the filters, where the filters of extraneous subunits tend to be driven to zero. The procedure of testing a series of NIMs with different subunit compositions can again be substantially facilitated by optimizing the filters in a low-dimensional stimulus subspace, such as identified by STC or GQM analysis (Fig. S5).

### RGC simulation details

In order to simulate the response of an ON-OFF RGC, we generated a Gaussian white noise process sampled at 15 Hz (such as a luminance-modulated spot stimulus), which was then filtered using separate ON- and OFF-like filters (Fig. 1A). These filter outputs were then rectified using functions of the form *f*(*x*) = log(1+exp(*b_1_*x)), summed together and the resulting signal was passed through a spiking nonlinearity of the form *F*[*x*] = *a*log(1+exp(*b_2_*(*x*-*c*))). This conditional intensity function was then used to generate a set of spike times. To generate heavy-tailed stimulus distributions (Figs. 3G, H), we sampled white noise from a Student's *t*-distribution with a range of values for the degrees of freedom to control the tail thickness.

The data were simulated at a temporal resolution of 8.3 ms, and model filters were represented at a lower resolution of 33 ms, with a length of 1 s. For the GQM and NIM we incorporated smoothness regularization on the filters, and for the NIM we also incorporated smoothness regularization on the upstream nonlinearity coefficients *a_ij_*.

To identify the STA/STC subspace depicted in Figs. 1 and 3, we performed STC analysis after projecting out the STA. For comparison with the NIM, we also created a simple model based on the STA and STC filters, using a GLM-based optimization of linear coefficients on the outputs of the STA filter and the squared outputs of the STC filters, similar to previous work [58]. Note that in order to maximize performance when estimating STC-based models, we did not project out the STA before computing the STC filters.

### LGN neuron model fit details

Data for the LGN example were recorded extracellularly from an anaesthetized and paralyzed cat by the Alonso Lab [45], [75], [76]. The stimulus consisted of 800 seconds of a 32×32 pixel natural movie, refreshed at 60 Hz, which was recorded from a camera mounted on top of a cat's head [100]. A 17×17 pixel patch of the movie was cropped around the receptive field, detected via STA at the optimal latency, and the movie was up-sampled by a factor of six, to produce a temporal resolution of 2.8 ms. Ten-fold cross-validation was used for evaluating model performance.

Each filter was represented by space-time separable center and surround components, and thus consisted of two sets of spatial and temporal filters [101]. Temporal filters were represented with 30 equally spaced tent basis functions, with grid points ranging from 0 to −240 ms. For the LN model, the spatial filters were initialized as Gaussian functions with the same center as the STA and different widths (1 pixel for the center and 6 pixels for the surround). For the GQM and NIM, both excitatory and suppressive filters were initialized to be the same as the optimal linear filters. In the filter optimization stage, the spatial and temporal filters were optimized alternately until convergence of the log-likelihood. Both the GQM and the NIM were fit using smoothness regularization for the spatial and temporal kernels, and sparseness regularization for the spatial kernels. For the NIM, we also used smoothness regularization on the *a_ij_* when estimating the upstream nonlinearities.

### Songbird auditory midbrain model fit details

Data for the songbird auditory midbrain example were provided by the Theunissen lab through the CRCNS database [83], and details of experimental methods can be found in [81], [82]. The example neuron was recorded extracellularly from the zebra finch mesencephalicus lateralis dorsalis (MLd). Stimuli consisted of 20 different conspecific bird songs, each lasting 2–4 sec, and each presented 10 times. These 20 songs were then divided into 5 equal groups for five-fold cross-validation. The raw sound waveforms were preprocessed by computing the spectrogram using a short-time Fourier transform to produce a stimulus matrix X(*t*,*f*), representing the power of the audio signal at frequency *f* and time *t*. We used a time resolution of 2 ms, and 20 uniformly spaced frequency bins, ranging from 250 Hz to 8 kHz. For estimating spectrotemporal filters, we used 20 time lags. Thus, each filter was represented by 400 parameters. Filter estimates were regularized using sparseness and smoothness penalties, where the smoothness penalty utilized the spectrotemporal Laplacian (with equal weighting in the frequency and time dimensions).

### V1 simulation details

The simulated V1 neurons shown in Fig. 6 were constructed as LNLN models (Fig. 2C). The stimulus filters were spatial Gabor functions that were amplitude- and phase-modulated in time (i.e., direction-selective). Stimulus filters were identical up to a spatial translation, and were weighted by a spatial Gaussian envelope. The filter outputs were then passed through a set of static nonlinear functions (either *x* ^2^, or log(1+exp(*b_1_x*))), before being summed together, and passed through the spiking nonlinearity (again, of the form *a*log(1+exp(*b_2_*(*x*-*c*))) to generate a conditional intensity function. Spike times were simulated in response to binary random bar stimuli [57], using a time resolution of 10 ms, and 24 bar positions.

Both the GQM and NIM were fit using a sparseness penalty on the filters. For the NIM, we also used smoothness regularization on the *a_ij_* when estimating the upstream nonlinearities. To measure how well the estimated model filters matched the true filters, we represented the model filters as linear combinations of the true filters.

### V1 neuron modeling details

The V1 neuron shown in Fig. 7 was recorded from an anesthetized macaque [57]. The stimuli (refreshed at 100 Hz) consisted of random arrays of black and white bars covering the neuron's classical receptive field, and oriented along its preferred orientation. Full experimental details can be found in [57]. Spatiotemporal filters were represented by 16 ‘pixels’ and 14 time lags. For model evaluation we used ten-fold cross-validation. Model fitting was analogous to that described for the V1 simulations (Fig. 6). STC-based models were constructed as described above for the simulated ON-OFF RGC.

The V1 neuron shown in Fig. 8 was recorded from an anesthetized cat [91]. The stimuli consisted of natural and naturalistic movies at various contrasts, including noise processes with pink spatial and white temporal statistics, pink temporal and white spatial statistics, pink temporal and pink spatial statistics, and natural movies recorded with a ‘cat cam’ [100]. The mean luminance across all stimuli (15 different stimuli, each lasting 2 minutes) was the same. The raw movies were 64×64 pixels and were sampled at 50 Hz. These raw movies were spatially down-sampled and cropped to produce 20×20 pixel patches that were individually mean-subtracted. Model performance was evaluated using five-fold cross-validation, with cross-validation sets constructed by taking 20% of the data from each stimulus type.

For all analysis we used 8 time lags to construct spatiotemporal filters (each described by 8×20×20 = 3200 parameters). For STA/STC analysis we first whitened the stimulus by rotating into the principal component axes and normalizing each dimension to have unit standard deviation [9]. Because the stimulus covariance matrix for natural stimuli has many eigenvalues close to zero, we avoided amplifying noise associated with these low-variance dimensions by using a pseudoinverse of the covariance matrix, effectively discarding the *n* lowest variance dimensions of the stimulus [9], [95]. In addition to removing biases due to pairwise correlations in the stimulus, this method effectively imparts a prior favoring spatiotemporally smooth filters, since the lowest variance dimensions of natural stimuli have high spatial and temporal frequencies. We retained 500/3200 of the stimulus dimensions for STA/STC analysis.

To estimate filters of the LN model, GQM and NIM, we used sparseness regularization, as well as penalties on the (two-dimensional) spatial Laplacian at each time lag. To display the three-dimensional spatiotemporal filters we plot the time slice of each filter containing the most variance across pixels (‘best time slice’), as well as the projection of the filter onto a spatial axis orthogonal to the neuron's preferred orientation (‘space-time projection’) [62]. The preferred orientation was determined by fitting a two-dimensional Gabor function to the best time slice for each filter, and taking the (circular) average of the individual Gabor orientations across all filters.

## Supporting Information

Figure S1 **Robustness of filter estimation.** **A**) For the simulated ON-OFF RGC in Figs. 1 and 3, the likelihood function with respect to the NIM stimulus filters shows only a single global optimum (up to an interchange of the filters) over a broad range of parameter space. To illustrate this, 100 iterations of the optimization were performed with random initializations of the filters, and in all cases the correct filters were identified. The initial filters are projected onto the true ON and OFF filters (inset), and are plotted along with the resulting optimized filter projections. Each iteration of the optimization is thus represented by a pair of optimized filters (large blue and red circles), along with a pair of initial filters (small blue and red circles, color coded based on the resulting filter estimates). **B**) For the example MLd neuron in Fig. 5, we found two distinct local maxima when optimizing the NIM stimulus filters, corresponding to the two clusters of the maximized log-likelihood across many repetitions of optimizing the filters with random initial conditions. The global optimum (right) corresponds to the set of filters shown in Fig. 5, while a locally optimum solution (left) corresponds to the excitatory filter matching the STA. **C**) For the simulated V1 neuron shown in Fig. 6A, optimization of the NIM is again well-behaved. In this case there are potentially several spurious local maxima, illustrated by the distribution of maximized log-likelihood values. However, these local maxima correspond to models that are very similar to the identified global maximum, as shown by the similar log-likelihood values, as well as the similarity of the identified filters (example models shown at left and right).
(EPS)

Figure S2 **NIM parameter optimization scales approximately linearly.** **A**) The time required to estimate the filters (black) and upstream nonlinearities (red) scales linearly as a function of data duration for the ON-OFF RGC simulation (with two subunits) shown in Figs. 1 and 3. The error bars show +/−1 standard deviation around the mean across multiple repetitions of the parameter estimation (with random initialization). Estimation was performed on a machine running Mac OS X 10.6 with two 2.26 GHz quad-core Intel Xeon processors and 16 GB of RAM. **B**) To measure parameter estimation time as a function of the number of stimulus dimensions, we simulated a V1 neuron (similar to that shown in Fig. 6A) receiving two rectified inputs (data duration of 10^5^ time samples). We then varied the number of time lags used to represent the stimulus and measured the time required for parameter estimation. Estimation of the stimulus filters scales roughly linearly with the number of stimulus dimensions, while estimation of the upstream nonlinearities is largely independent of the number of stimulus dimensions. **C**) Parameter estimation time for the filters and upstream nonlinearities also scales approximately linearly as a function of the number of subunits. Here we again used a simulated V1 neuron similar to that shown in Fig. 6A, although with 10 rectified inputs (200 stimulus dimensions and data duration of 10^5^ time samples). Note that the additional step of estimating the upstream nonlinearities adds relatively little to the overall parameter estimation time, especially for more complex models.
(EPS)

Figure S3 **Comparison of the NIM and GQM for the example LGN neuron.** The linear model (**A**), NIM (**B**), and GQM (**C**) fit to the example LGN neuron from Fig. 4 are shown for comparison. Here (A) and (B) are reproduced from Figs. 4A and B respectively. Note that the spatial and temporal profiles of the linear and squared (suppressive) GQM filters are largely similar to the (rectified) excitatory and suppressive filters identified by the NIM. Despite the similarity of the identified filters, however, the NIM and GQM imply a different picture of the neuron's stimulus processing, as illustrated in Fig. S4.
(EPS)

Figure S4 **Different predictions of the GQM and NIM with excitation and delayed suppression.** The GLM (**A**), NIM (**B**), and GQM (**C**) fit to the example MLd neuron in Fig. 5 (A and B here are reproduced from Figs. 5A and B). The NIM and GQM identify similar excitatory and suppressive filters, but the GQM assumes linear and squared upstream nonlinearities for these inputs respectively, while the NIM infers the rectified form of these functions. Despite the similarities in the identified filters, the different upstream nonlinearities in these models imply distinct interactions between the excitatory and suppressive inputs. To illustrate this, we consider how these different models process two stimuli in (D) and (E), which highlight these differences. **D**) First, we consider a negative impulse (left) presented at the preferred frequency (horizontal black lines in A–C). The outputs of the excitatory (blue) and suppressive (red) subunits are shown for the linear model (top), GQM (middle), and NIM (bottom). The combined outputs of these subunits are then transformed by the spiking nonlinearity into the corresponding predicted firing rates at right. In this case, only the linear model responds to the stimulus, since the GQM is strongly suppressed, and the NIM is largely unaffected due to the rectification of the negatively driven inputs. **E**) Similar to (D), we consider a biphasic stimulus (left), also presented at the neuron's preferred frequency. This stimulus drives different responses in all three models. The response predicted by the GQM is by far the weakest because the (squared) suppression driven by the initial negative phase of the stimulus coincides with the excitation driven by the positive phase of the stimulus, causing them to partially cancel each other out. For the NIM, the negative phase of the stimulus does not drive the suppression, due to rectification, and the excitation is able to elicit a much larger response. The response predicted by the linear model is even larger since this is essentially the optimal stimulus for driving the linear filter. This suggests targeted stimuli that might be able to distinguish the computations being performed by MLd neurons.
(EPS)

Figure S5 **Selecting the number of model subunits.** **A**) To illustrate the robustness of NIM parameter estimation to specification of the precise number of subunits, we first consider the simulated V1 neuron from Fig. 6A, which was constructed from six rectified excitatory subunits. Fitting a sequence of NIMs (blue) and GQMs (red) with increasing numbers of (excitatory) subunits reveals that the log-likelihood (evaluated on a simulated cross-validation data set) initially improves dramatically, but becomes nearly saturated for models with four or more subunits. Here we plot log-likelihood relative to that of the best model, and error bars show one std. dev. about the mean. While it is possible in this case to identify the true number of underlying subunits (six) from the cross-validated model performance of the NIM, the model performance is relatively insensitive to specification of the precise number of subunits. **B**) Stimulus filters from example NIM fits from (A), with four, six, and eight filters. Note that the identified filters are nearly identical across these different models, and when more than the true number (six) of subunits are included in the model, sparseness regularization on the filters tends to drive the extra filters to zero, yielding effectively identical models. **C**) To illustrate the procedure of selecting the number of model subunits with real data, we consider fitting a series of models to the example MLd neuron from Fig. 5. In this case there are both excitatory and suppressive stimulus dimensions, so we independently vary the number of each. Average (+/−1 std. error) cross-validated model performance is depicted for each subunit composition for models with up to three subunits (the color indicates the number of suppressive subunits). While we do not have sufficient data to identify statistically significant differences, a two-filter model with one excitatory and one suppressive filter appears to achieve optimal performance. **D**) Three example NIM fits for one, two, and three-filter models corresponding to the Roman numerals in (C). (i) Model with one excitatory subunit. (ii) Model with one excitatory and one suppressive subunit. (iii) Model with two excitatory and one suppressive subunits. Note that the excitatory and suppressive filters from the two-filter model are also present in the three-filter model, and that the addition of a second excitatory subunit (resembling the linear filter) provides little, if any, additional predictive power. **E**) Similar to (C–D), we consider fitting models with different numbers of excitatory and suppressive subunits to the example macaque V1 neuron from Fig. 7. In this case, the neuron is selective to a large number of stimulus dimensions (Fig. 7A), and thus there are a large number of possible excitatory/suppressive subunit compositions to consider. To greatly speed this process (and illustrate a procedure for rapid model characterization), we fit the NIM filters in a reduced stimulus subspace (see Methods) that is identified by a GQM with four excitatory and six suppressive dimensions. The number of subunits in the GQM was selected in order to ensure that all filters with discernible structure were included. The figure then shows the average (+/−1 std. error) cross-validated log-likelihood (relative to a model with two excitatory and one suppressive filters) for NIMs with varying numbers of excitatory and suppressive subunits. Note that the model performance increases initially, but tends to saturate for models with more than about four excitatory and four suppressive subunits. For comparison, the cross-validated log-likelihood of the GQM (green line) – which established the stimulus subspace – is below most of the NIM solutions. While fitting the stimulus filters in the full stimulus space provides slightly different (though qualitatively very similar) results, limiting the NIM to the subspace provides a tractable way to fully explore the nonlinear structure of computation, and can then serve as an initial guess for a more computationally-intensive search in the full stimulus space. **F–G**) Two example NIM fits from those depicted in (E). A NIM with four excitatory and four suppressive subunits (F) is compared to a NIM with six excitatory and six suppressive subunits (G), the latter providing only a slight improvement relative to the former. Both models provide a qualitatively similar depiction of the neuron's stimulus processing, identifying largely similar sets of excitatory and suppressive inputs.
(EPS)

Figure S6 **Selection of regularization parameters.** To illustrate how the performance of our models depends on selection of the regularization hyperparameters, we fit a series of models to the example V1 neuron from Fig. 8. For this example neuron regularization of the stimulus filters is particularly important, given the large number (3200) of parameters associated with each filter. As described in the Methods section, we use both smoothness (L2 penalty on the spatial Laplacian) and sparseness regularization on the filters, each of which is governed by a hyperparameter. While in principle we could independently optimize these regularization parameters for each filter, we consider here only the case where all filters are subject to the same regularization penalties. Further, we consider optimizing the smoothness and sparseness penalties independently, which will not in general identify the optimal set of hyperparameters. **A**) We first set the sparseness regularization penalty to zero, and systematically vary the strength of the smoothness penalty. The cross-validated log-likelihood is plotted for the NIM (blue trace) and GQM (red trace), showing that the NIM outperforms the GQM over a range of smoothness regularization strengths. **B–D**) Representative filters are shown from model fits at several regularization strengths, as indicated by the black circles in (A). The filters are depicted as the ‘best-time slice’ (BTS) and the ‘space-time projection’ (STP), as in Fig. 8. **E**) Similar to (A), we next consider varying the strength of sparseness regularization given fixed values for the smoothness regularization (set to the value indicated by the vertical dashed line in A). Note that the performance of the NIM again remains significantly better than the GQM across a range of regularization strengths. **F–H**) Representative filters at several sparseness regularization strengths, as indicated in (E). Note that (F) is identical to (C), reproduced for ease of comparison.
(EPS)
